# Early‐life single‐episode sevoflurane exposure impairs social behavior and cognition later in life

**DOI:** 10.1002/brb3.514

**Published:** 2016-07-04

**Authors:** Daisy Lin, Jinyang Liu, Lea Kramberg, Andrea Ruggiero, James Cottrell, Ira S. Kass

**Affiliations:** ^1^ Anesthesiology DepartmentSUNY Downstate Medical CenterBox 6, 450 Clarkson AveBrooklynNew York11203; ^2^ Department of Physiology and PharmacologySUNY Downstate Medical Center450 Clarkson AveBrooklynNew York11203; ^3^ The Robert F. Furchgott Center for Neural and Behavioral SciencesBrooklynNew York11203

**Keywords:** Cognition and social interaction, postnatal day 7, sevoflurane

## Abstract

**Background:**

Single‐episode anesthetic exposure is the most prevalent surgery‐related incidence among young children in the United States. Although numerous studies have used animals to model the effects of neonatal anesthetics on behavioral changes later on in life, our understanding of the functional consequences to the developing brain in a comprehensive and clinically relevant manner is unclear.

**Methods:**

The volatile anesthetic, sevoflurane (sevo) was administered to C57BL6 postnatal day 7 (P7) mice in a 40% oxygen and 60% nitrogen gas mixture. In order to examine the effects of sevo alone on the developing brain in a clinically relevant manner, mice were exposed to an average of 2.38 ± 0.11% sevo for 2 h. No sevo (control) mice were treated in an identical manner without sevo exposure. Mice were examined for cognition and neuropsychiatric‐like behavioral changes at 1–5 months of age.

**Results:**

Using the active place avoidance (APA) test and the novel object recognition (NOR) test, we demonstrated that P7 sevo‐treated mice showed a deficit in learning and memory both during periadolescence and adulthood. We then employed a battery of neuropsychiatric‐like behavioral tests to examine social interaction, communication, and repetitive behavior. Interestingly, compared to the no‐sevo–treated group, sevo‐treated mice showed significant reductions in the time interacting with a novel mouse (push–crawl and following), time and interaction in a chamber with a novel mouse, and time sniffing a novel social odor.

**Conclusions:**

Our study established that single‐episode, 2‐h sevo treatment during early life impairs cognition later on in life. With this approach, we also observed neuropsychiatric‐like behavior changes such as social interaction deficits in the sevo‐treated mice. This study elucidated the effects of a clinically relevant single‐episode sevo application, given during the neonatal period, on neurodevelopmental behavioral changes later on in life.

## Introduction

General anesthesia has been used in young children during surgical procedures dating back as early as the 1800s (Costarino and Downes 2005; Mai and Cote 2012). Currently, an annual estimate of over 1 million children under the age of 4 received medical procedure‐related anesthesia in the United States (Rabbitts et al. 2010). However, it is only in the last decade that we started to recognize that general anesthesia has deleterious effects on the developing brain (Jevtovic‐Todorovic et al. 2003; Fredriksson et al. 2007; Mellon et al. 2007; Slikker et al. 2007; Loepke and Soriano 2008; DiMaggio et al. 2009, 2011; Wilder et al. 2009; Brambrink et al. 2010; Sun 2010; Stratmann 2011; Amrock et al. 2015). With arising concerns regarding the safe use of general anesthesia in young children, there is an urgent demand to clearly understand the resulting functional changes in the brain in a comprehensive and clinically relevant manner.

This current study investigates the resulting functional changes that have been widely established, such as cognition, as well as those still unknown, such as neuropsychiatric disorders. Using the mouse as a model system, we examined these functional changes while maintaining a close relevance of our study approach to clinical settings. Our focus is to examine the implications of single‐episode anesthetic exposure on early brain development because it is the most prevalent surgery‐related incidence among children under the age of 4 (Wilder et al. 2009).

Numerous human and animal studies had been conducted to examine the association between early‐life general anesthetic exposure and behavioral changes later on in life, with a focus on cognitive function (Jevtovic‐Todorovic et al. 2003; DiMaggio et al. 2009; Sprung et al. 2009; Wilder et al. 2009; Flick et al. 2011; Murphy and Baxter 2013; Shen et al. 2013a). A majority of the human studies were conducted with a retrospective population cohort approach, by gathering data from a specific subpopulation and identifying incidences of general anesthetic exposure before the ages of 3–6. However, variations in these human study approaches such as different assessment tools have led to inconsistent conclusions; some groups reported cognitive impairment (DiMaggio et al. 2009, 2011; Sprung et al. 2009; Wilder et al. 2009), while others did not (Bartels et al. 2009; Kalkman et al. 2009; Hansen et al. 2011, 2013). Although prospective human studies have started to emerge in recent years, an agreement in outcome has yet to be established. An interim secondary outcome study from one group did not find increased risk of neurodevelopmental outcome at 2 years of age (Davidson et al. 2016), while another group found cognitive impairment in children ages 6–11 years (Stratmann et al. 2014).

Animal studies, ranging from rodents to nonhuman primates, have persistently showed an association between early‐life general anesthetic exposure and deficits in learning and memory‐related behavior. However, anesthetics such as isoflurane (iso), sevoflurane (sevo), or ketamine were generally given in the range of 4–8 h in rodents (Jevtovic‐Todorovic et al. 2003; Sanders et al. 2008; Loepke et al. 2009; Satomoto et al. 2009; Stratmann et al. 2009; Liang et al. 2010; Murphy and Baxter 2013; Shen et al. 2013a; Wang et al. 2013; Lee et al. 2014a), to as long as 5–24 h in monkeys (Zou et al. 2009, 2011; Brambrink et al. 2010; Paule et al. 2011). As a comparison, children undergoing routine surgeries are typically exposed to only 1 MAC (minimum alveolar concentration) of iso or sevo for <1 h (Rabbitts et al. 2010). Therefore, the reported behavior changes as a result of anesthetic exposure in animals have not accurately depicted the effects of anesthetics on the developing brains of young children.

The developing brain is vulnerable to a variety of environmental insults, ranging from deprivation of maternal care to toxin and drug exposure, resulting in increased risk of neuropsychiatric disorders, such as autism spectrum disorder, depression, anxiety, bipolar disorder, schizophrenia, and obsessive compulsive disorder (Pichot 1986; Ansorge et al. 2004; Batten et al. 2004; Phillips et al. 2005; Read and Hammersley 2005; Cath et al. 2008; Champagne and Curley 2009; Roullet et al. 2013; Lee et al. 2015). Exposure to anesthetics during a critical developmental time period is a major environmental insult to the brain. However, the effect of early‐life exposure to anesthetics on the development of neuropsychiatric disorders is not clear and therefore is investigated as an area of functional changes in this current study.

We investigated the role of early‐life exposure to sevoflurane on cognition and neuropsychiatric‐like behavioral changes. Sevo is the most commonly used volatile anesthesia for surgical procedures on both children and adults in the United States (Sakai et al. 2005). By exposing postnatal day 7 (P7) mice to a single episode of sevo for 2 h, we established that cognitive ability was impaired later on in life. Interestingly, we also observed with three different behavior paradigms that early‐life exposure to sevo resulted in social deficits. This study extends our awareness of the insults that single‐episode exposure to sevo has on the developing brain, resulting in long‐lasting functional changes that we can observe through behavior later on in life.

## Materials and Methods

### Treatment with sevoflurane

C57/BL6 mice were used throughout the study, which was approved by the SUNY Downstate IACUC. A total of nine litters of mice were used to establish the approximate MAC for P7 mice. A separate set of 11 litters of mice were used for treatment without tail clamp and mice from this group were used for behavioral tests later on in life. At P7, all male pups from each litter (ranging from 2 to 6 pups) were randomly assigned to either the sevo or the no sevo (control) group, while the female pups remained with the dam. During a 2‐h treatment period, pups from the sevo group were separated from the dam and exposed to sevo in a 40% oxygen (O_2_) and 60% nitrogen (N_2_) gas mixture (GTS‐WELCO, Newark Distribution, Morrisville, PA). These pups were placed on a 37°C heating pad to prevent hypothermia during treatment. A pulse oximeter sensor (MSTAT 4 mm, Kent Scientific Corporations, Torrington, CN) was placed on one of the hind paws of the pup and measurements for heart rate (HR) and blood oxygen saturation (SpO_2_) were recorded every 5 min. To establish the approximate MAC of sevo on P7 mice, each treatment of sevo consisted of two mice and tail clamp was done every 10 min. The sevo concentration was adjusted to a higher concentration if both mice moved during tail clamp, adjusted to a lower concentration when neither mouse moved during tail clamp, and no adjustment was made when only one mouse responded to stimuli. This was our approach to determine that with a given sevo concentration, 50% of mice did not respond to stimuli. The sevo concentration was recorded every 5 min since that was the time interval that we used to record peripheral capillary oxygen saturation (SpO_2_) and HR. The pups from the control group were also separated from the dam and exposed only to 40% O_2_ and 60% N_2_. At the end of the 2‐h treatment the pups were returned to their home cage and reunited with their dams. All pups were then reared and weaned following standard institution procedures.

### Behavior tests

The mice that were involved in the behavior tests had undergone a 2‐h sevo or no sevo treatment at P7 without tail clamping. They were reared and group housed under standard conditions. The sevo‐treated mice were marked to distinguish them from the no‐sevo–treated mice within a litter. We examined at most one to two litters of mice at a time for each behavior, with at least 1 week of resting time in between different behavioral tests. The behavior tests were given sequentially for the active place avoidance (APA), reciprocal social interaction, and olfaction habituation/dishabituation. After completion of these tests, we then introduced three‐chamber interaction, open field, and novel object recognition (NOR). All behavioral apparatus were assembled and remained in their original locations throughout the entire duration of the project. The APA test was done on mice starting at the age of P27. All other behaviors were conducted on mice within the age range of 1.5–5 months old. The following reasons contributed to variation in the number of mice used for some tests. First, we were not able to examine all treated mice on the APA due to irreparable malfunctioning of the APA apparatus. Therefore, we introduced NOR as a second cognition test on mice that had not been used for the APA. Second, some mice were not included in testing if they were not within the age range at the time of the test, specifically the second group of tests such as three‐chamber interaction, open field, and NOR. Besides the APA and the open field, all other tests that required manual scoring were first videotaped and then scored by experimenters who were blind to the treatment status of the mice.

### Locomotion and anxiety‐like behavior

#### Open field test

An open field apparatus was used to assess the general physical and anxiety‐like performance of the mice based on their ambulatory locomotion in the arena (Crawley 1985). In a well‐lit novel room, each mouse was given 30 min to explore the open field arena. Locomotion activities such as distance and time in different compartments of the arena were automatically measured using a computerized tracking apparatus (Versadat, Versamax, Groovy, CA).

### Learning and memory‐like behavior

#### Active place avoidance test

The APA test is a hippocampus‐dependent spatial memory test. A rotating arena consisting of a circular platform (40 cm diameter) was placed in the center of a dimly lit room. The mouse was trained to avoid a 60° shock zone, which could be defined within a region of the room identified by multiple visual cues (Fenton et al. 1998; Wesierska et al. 2005). Two‐day trials were performed as described previously (Burghardt et al. 2012). Briefly, the mouse was given 10 min for each trial with at least a 50‐min intertrial interval. The locomotion of the mouse was tracked by computer‐based software that analyzed images from an overhead camera and delivered shocks appropriately (Tracker, Bio‐Signal Group Corp., Brooklyn, NY). A brief constant current foot shock (500 msec, 60 Hz, 0.2 mA) across pairs of rods was delivered to the shock zone upon entrance of the mouse. Track analysis software (Bio‐Signal Group Corp.) was used to compute the number of times that the mouse entered the shock zone.

#### Novel object recognition test

This is a two consecutive day test examining learning and memory‐like behavior on adult male mice (3–5 months of age; Leger et al. 2013). The test was conducted in a room with dim lighting. Day 1 is considered the familiarization phase. Mice were individually habituated in a standard open field apparatus for 10 min. They were then taken out of the arena briefly and two identical glass bottles filled with pink silica gel were placed in the center of the arena. The glass bottles were positioned 5 inches from each other such that the mouse can travel freely across the center of the arena without obstruction. The mouse was then put back in the arena and allowed 10 min to become familiar with the two identical objects. Day 2 is the test phase. One of the glass bottles is taken out of the arena and replaced with a yellow laboratory tube rack (H 6.5, W 3.5, D 2 inches) as a novel object. The holes on the sides of the tube rack were taped to prevent the mouse from climbing on them during the experiment. The same mouse was placed in the arena for 10 min in an identical manner as day 1 and allowed 10 min of exploration time. The times spent sniffing and interacting with (attempting to climb up or jump on or at) the familiar and the novel objects were scored for each mouse.

### Social interactions

#### Reciprocal social interaction

The subject mouse (P7 sevo treated or no sevo control) was transferred from his home cage to a new cage with fresh bedding and allowed to habituate to the cage for 10 min. At the end of this 10‐min session, a novel male target mouse (that had not undergone treatment during P7) of similar age was introduced into the same cage. The subject and the target mice were allowed to interact for 10 min. The amount of time that the subject mouse spent interacting with the target mouse (push–crawl/following behavior), self‐grooming, and exploring the arena and the total time the mouse was mobile were scored manually (Silverman et al. 2010).

#### Three‐chamber interaction

A three‐chamber apparatus made of clear plexiglass was used for this study (Nadler et al. 2004). The apparatus is divided into three equally sized compartments (H 9.5, W 8, D 16 inches). First, the subject mouse was habituated for 10 min in the center chamber. Then the doors that give access to the left and right sides of the chamber were opened allowing the subject mouse to freely explore all three chambers for 10 min. During this time, the novel target mouse was habituated under a wire pencil cup on a separate tabletop. After 10 min of three‐chamber exploration, the doors were closed and the subject mouse was briefly confined in the center chamber. During this time, we set up the three‐chamber apparatus such that the novel target mouse was placed on one side of the chamber and a novel empty pencil cup on the other side. A weighted plastic cup was placed on the top of each pencil holder to prevent the subject mouse from climbing on the top of it. The doors were then opened to allow the subject mouse to explore the three chambers for 10 min.

### Communication

#### Olfaction habituation/dishabituation

The mouse was transferred to a new cage containing a thin layer of fresh bedding and a hole for inserting a cotton tipped swab. After a 10‐min habituation period in the new cage, the mouse was presented with nonsocial and social odors. Each odor was presented for three consecutive times; the order of presentation was water, almond extract (1:100, Spice Supreme), orange extract (1:100, McCormick), mouse socials 1 and 2. The mouse social odors were taken by wiping in a zigzag pattern across the bottom surface of different cages for odors 1 and 2; each cage housed unfamiliar mice of the same sex and strain. Each presentation of odor lasted 2 min. The amount of time that the mouse spent sniffing the cotton swab, including nose poking, chewing, sniffing, and close proximity (2 cm) of the nose to cotton swab was scored (Silverman et al. 2010).

### Statistical analysis

All statistical analysis was done using GraphPad Prism 5.0 (GraphPad, San Diego, CA). Data with one variable such as the open field and the reciprocal social interaction were analyzed by *t* test. Data with two variables such as the APA, the NOR, the three‐chamber interaction, and the olfaction habituation/dishabituation were analyzed by two‐way ANOVA, followed by Bonferroni posttests.

## Results

### Establishing a mouse model of neonatal sevoflurane treatment

**Figure 1:**
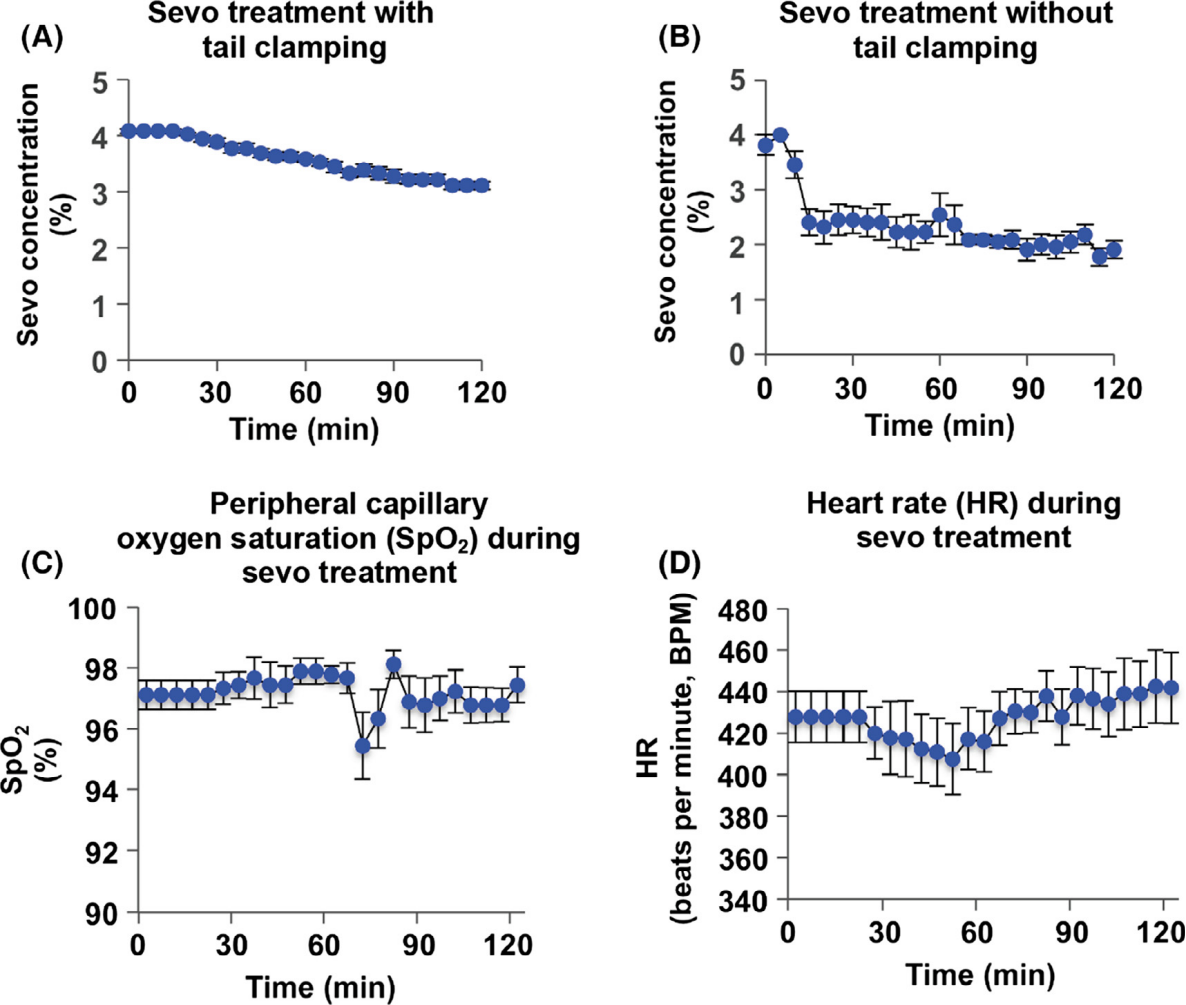
Two approaches of sevo treatment on postnatal day 7 (P7) mice. (A) The approximate minimum alveolar concentration (MAC) of sevo necessary for P7 mice was established by tail clamping every 10 min during treatment. The approximate MAC of sevo was averaged to be 3.58 ± 0.07% based on the concentration recorded every 5 min (*N* = 13). (B) On a separate group of mice, less than one MAC (2.38 ± 0.11%) of sevo was given without tail clamping. This group of mice was then used in all the subsequent behavior paradigms (*N* = 17). (C and D) Data shown are measurements taken from the group of mice that were exposed to sevo with tail clamp. SpO_2_ and heart rate were recorded every 5 min on all mice undergoing sevo treatment.

In order to understand the effect that neonatal sevo treatment alone has on behavioral changes later on in life, we established two different sevo treatment groups. During a 2‐h treatment period, we used tail clamp to first establish that the approximate MAC of sevo for P7 mice averaged 3.58 ± 0.07% (Fig. 1A). Since tail clamp may result in pain and scaring of the tail similar to the act of clinical surgery, we then treated a separate group of mice in a similar manner but without tail clamp. This second group of mice was treated with a reduced concentration of sevo, which was sufficient to keep the mice immobilized/unconscious and this sevo concentration averaged 2.38 ± 0.11% (Fig. 1B). These mice were subsequently examined for the effect of neonatal sevo treatment alone on behavioral changes later on in life. Mice were monitored closely for their measurements of peripheral capillary oxygen saturation (SpO_2_) and HR during the treatment. An average SpO_2_ of 97 ± 0.11% and HR of 427 ± 2.02 beats per min suggest the mice were in a physiological healthy state, without any signs of hypoxia (Fig. 1C and D).

### Locomotion and anxiety‐like behavior

**Figure 2:**
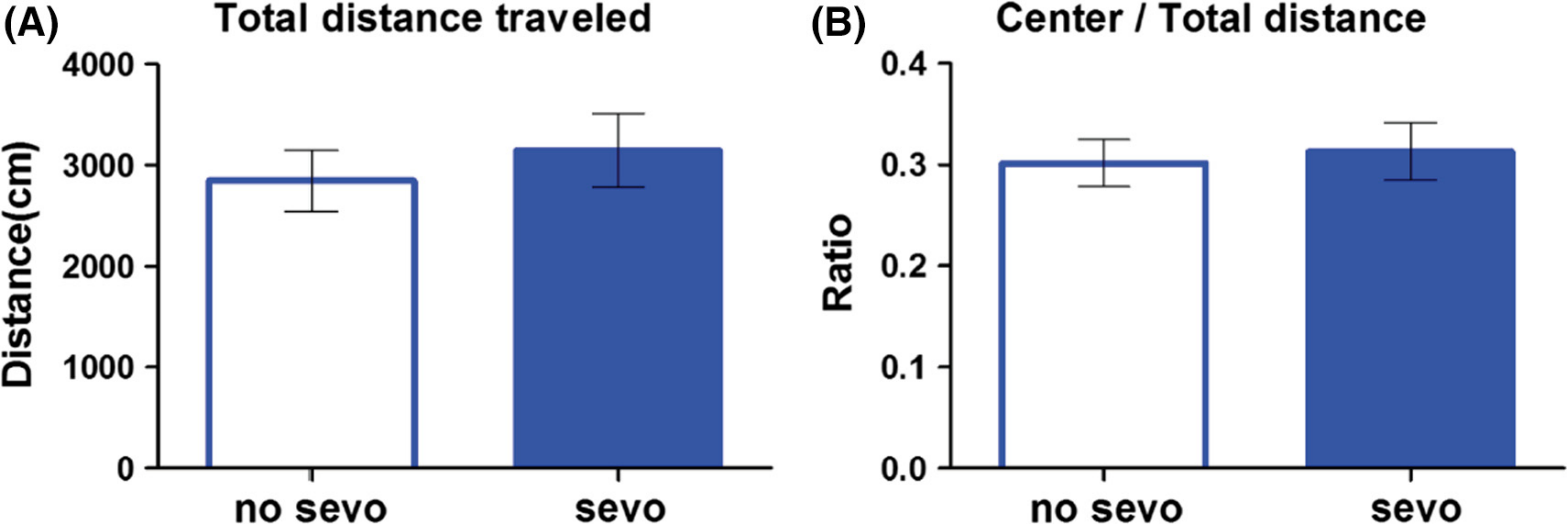
Postnatal day 7 sevo treatment did not have an effect on locomotion and anxiety‐like behavior later on in life. No differences were observed between the two groups (no sevo vs. sevo) on different measurements of the open field apparatus such as (A) total distance traveled and the (B) ratio of center/total distance traveled. Unpaired *t*‐test with Welch's correction was used for statistical calculation (*N* = 14 for no sevo; *N* = 13 for sevo).

Locomotion and movement of the limbs are critical to all mouse behavior. Therefore, the no–sevo‐ and sevo‐treated mice were examined for their locomotion in the open field apparatus as a general physical assessment (Crawley 1985, 2007; Fig. 2). This brightly lit, novel test environment with an unprotected center is also anxiety provoking. The two groups of mice were examined for their exploration in the center versus the total arena as a measurement of anxiety‐like behavior. We observed no differences between the two groups on locomotion (Fig. 2A) and anxiety‐like behavior (Fig. 2B; unpaired *t*‐test with Welch's correction). Data suggest that locomotion and anxiety‐like behaviors are not potential confounds to subsequent behavioral tests.

### Learning and memory

#### Active place avoidance

**Figure 3:**
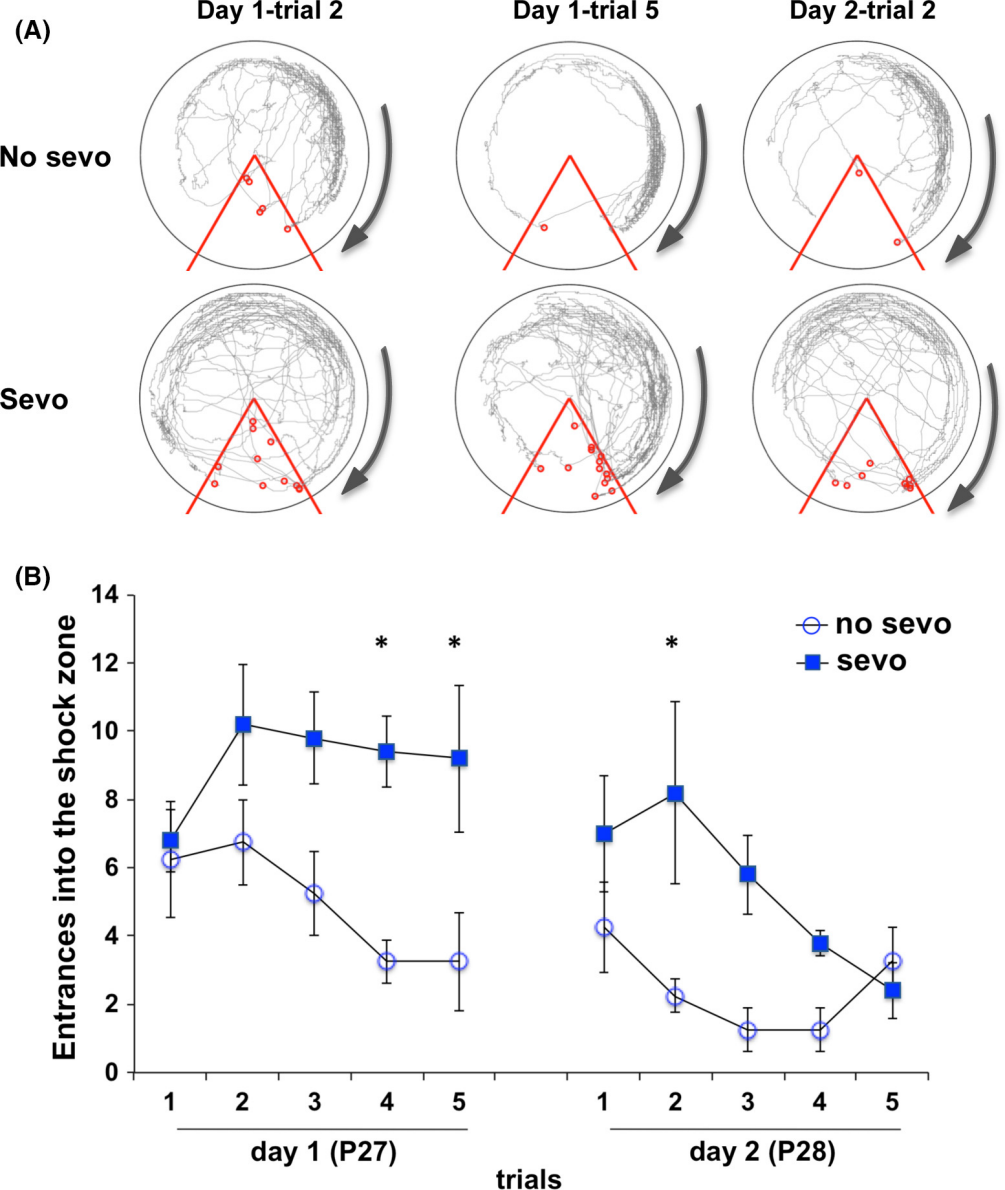
Neonatal sevo treatment impaired learning and memory during periadolescence. (A) Locomotion of a no‐sevo‐ and a sevo‐treated mouse are represented by traces on the circular rotating platform. The arrow indicates the direction of platform movement (1 revolution per minute). Entrances into the shock zone (represented by the 60° red zone) are marked as the red dots. (B) At P27 and P28, sevo‐treated mice entered the shock zone significantly more compared to the no‐sevo–treated group during a total of 10 trials. Two‐way ANOVA was used for statistical calculation: *F*_(1, 63)_ = 1.28, *P* < 0.01 for treatment; *F*_(9, 63)_ = 14.6, *P* < 0.001 for trials; *F*_(9, 63)_ = 1.8, *P* > 0.05 for interaction between treatment and trials. Bonferroni posttests showed *P* < 0.05 for treatment on day 1, trials 4 and 5; day 2, trial 2. Asterisk (*) denotes *P* < 0.05 for treatment effect within the trials (*N* = 4 for no sevo, *N* = 5 for sevo).

We observed learning and memory impairment as early as periadolescent age (P27 and P28), based on the hippocampus‐dependent APA test. The mouse learned to avoid a stationary shock zone in a constant rotating arena using the distal room landmarks as cues (Fig. 3). The no‐sevo–treated mice learned to avoid the shock zone over five trials during day 1 and this behavior persisted into day 2. A similar observation was not present in the sevo group. Sevo‐treated mice showed significantly more entrances into the shock zone over the 2 days, 10‐trial period (two‐way ANOVA followed by Bonferroni posttests, *P* < 0.01 for treatment, *P* < 0.001 for trial, *P* > 0.05 for interaction between treatment and trial, and *P* < 0.05 for treatment effect within trials, day 1—trials 4 and 5 and day 2—trial 2).

#### Novel object recognition

**Figure 4:**
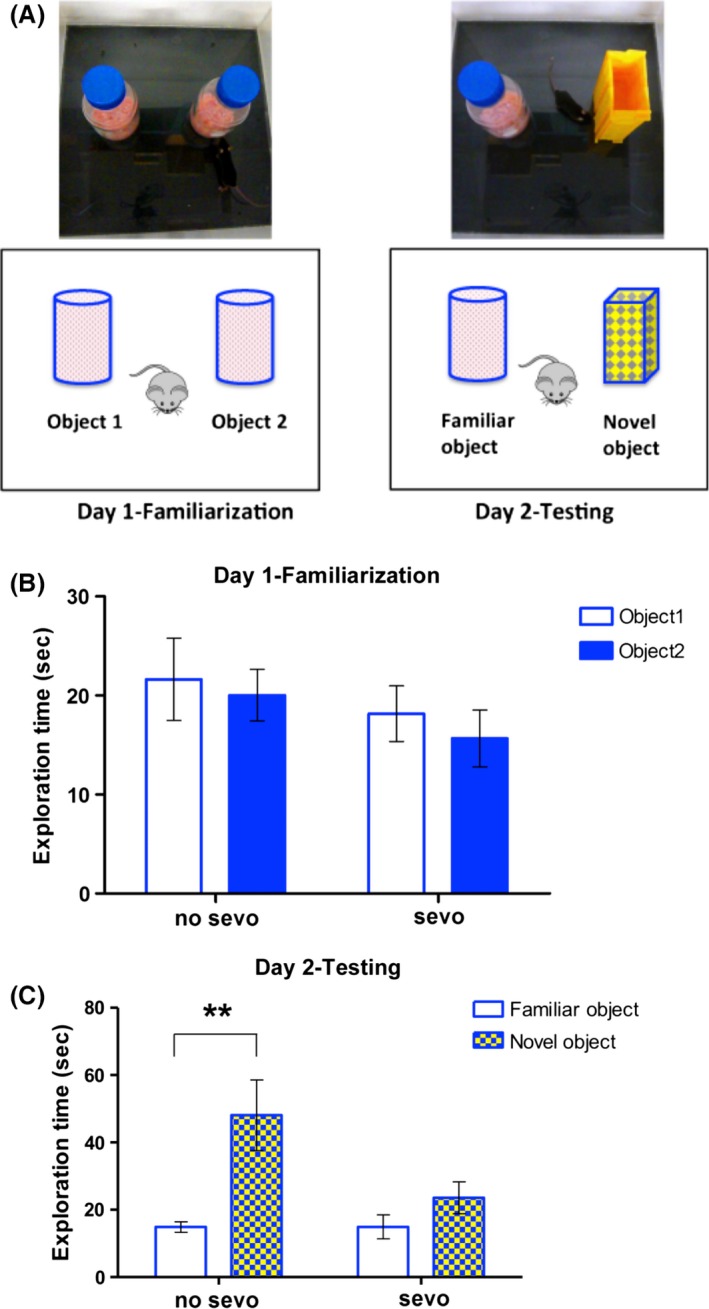
Neonatal sevo‐treated mice showed impairment in learning and memory during adulthood. (A) A schematic layout of the novel object recognition task that was used to examine the learning and memory behavior in mice. (B) During day 1—familiarization, both no sevo and sevo groups spent similar amounts of time exploring the two identical objects. No preference for a specific object was detected. A two‐way ANOVA was used for statistical analysis, *P* > 0.05 for treatment, object, and interaction between treatment and object. (C) During day 2—testing, while the no‐sevo–treated group spent significantly more time exploring the novel object, the sevo‐treated group did not show an increased interest. Two‐way ANOVA, followed by Bonferroni posttest were used for statistical analysis, *F*_(1, 36)_ = 3.6, *P* > 0.05 for treatment; *F*_(1, 36)_ = 10.4, *P* < 0.01 for object; *F*_(1, 36)_ = 3.6, *P* > 0.05 for interaction between treatment and object. Asterisk (**) denotes *P* < 0.01 for exploration time between the two different objects in the no sevo group (*N* = 11 for no sevo; *N* = 9 for sevo).

We examined cognitive function of adult mice (ages 4–5 months) by the NOR test. The NOR is a widely used learning and memory task, which offers no external stimuli or reinforcement (Leger et al. 2013). During day 1—familiarization (Fig. 4A), both the no‐sevo‐ and sevo‐treated groups spent a similar amount of time exploring the two identical objects, with no differential preference for a specific object (Fig. 4B; two‐way ANOVA). However, during day 2—testing (Fig. 4A), the no sevo group spent significantly more time exploring the novel object; this difference was not found in the sevo‐treated group (Fig. 4C; two‐way ANOVA, followed by Bonferroni posttests, *P* < 0.01 for time exploring the objects in the no sevo group). Combining the two sets of learning and memory behavioral tests, data demonstrated for the first time, a 2‐h, single‐episode neonatal exposure to less than one MAC of sevo impairs learning and memory as early as periadolescence and this persists to adulthood.

### Social interactions

#### Reciprocal social interaction

Mice are social animals that engage in a variety of social interaction behaviors. The reciprocal social interaction paradigm is designed to provide the most detailed and direct insight into how two unfamiliar mice interact in a standard new cage.

**Figure 5:**
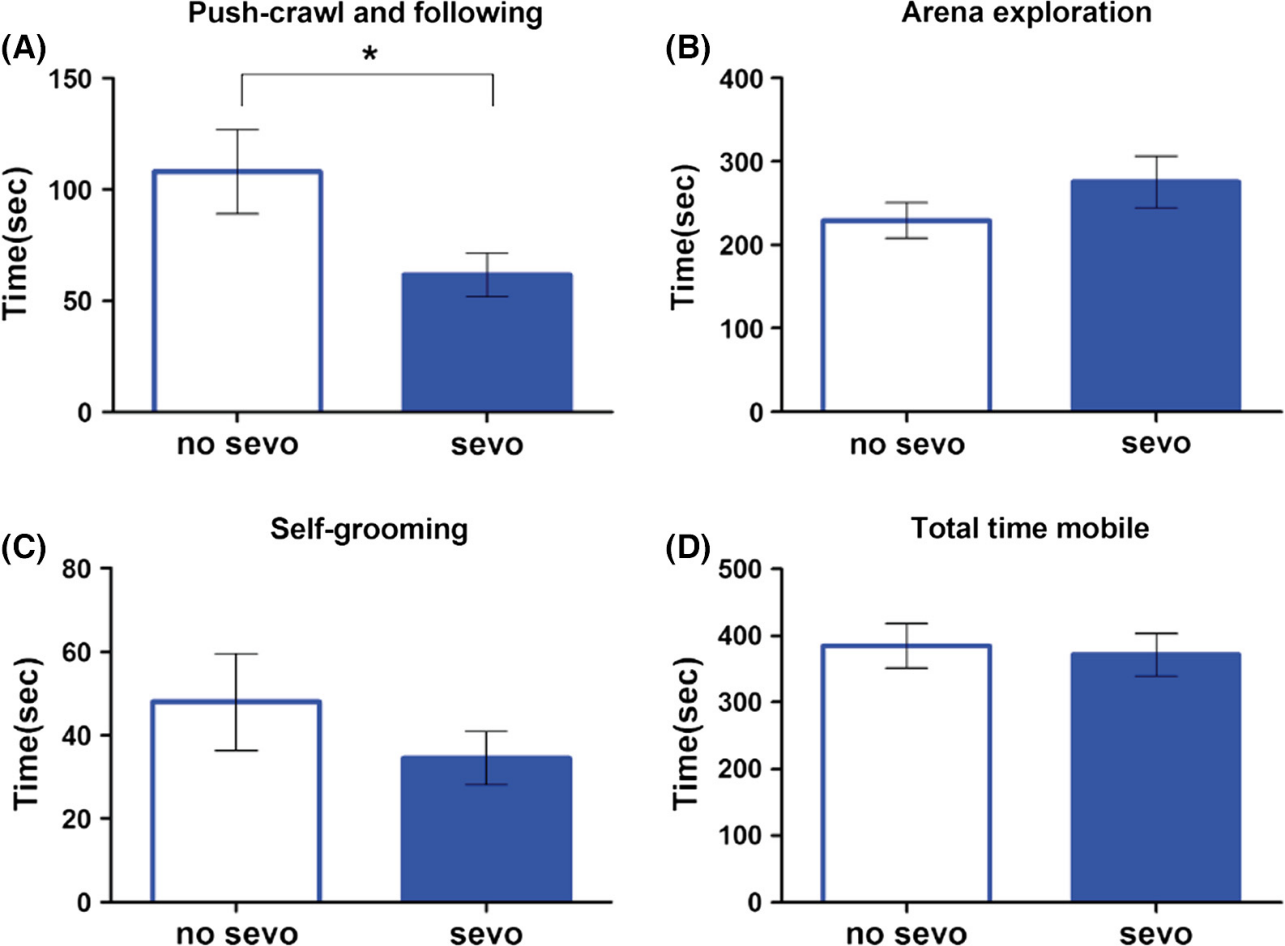
Neonatal sevo‐treated mice had a deficiency in social interaction behaviors as shown by reciprocal social interaction. (A) The sevo‐treated mice showed significantly less push–crawl and following toward a novel mouse of the same sex and similar age compared to the no‐sevo–treated mice. (B–D) During the 10 min of the reciprocal social interaction paradigm, the two groups of mice did not display a difference in arena exploration, self‐grooming, or total time being mobile in the cage. Unpaired *t*‐test with Welch's correction was used for statistical calculation. Asterisk (*) denotes *P* < 0.05 (*N* = 18 for no sevo; *N* = 17 for sevo).

We scored the four most predominant behaviors of the subject mouse while paired with the target mouse in the novel cage, such as push–crawl/following, arena exploration, self‐grooming, and time being mobile. Among the four measurements, sevo‐treated mice showed a specific deficit compared to no‐sevo–treated mice on the amount of time they engage in push–crawling and following the unfamiliar mouse (Fig. 5; unpaired *t*‐test with Welch's correction, *P* < 0.05). Data show mice exposed to single‐episode sevo treatment on P7 had impaired social interaction later on in life.

#### Three‐chamber social interaction

**Figure 6:**
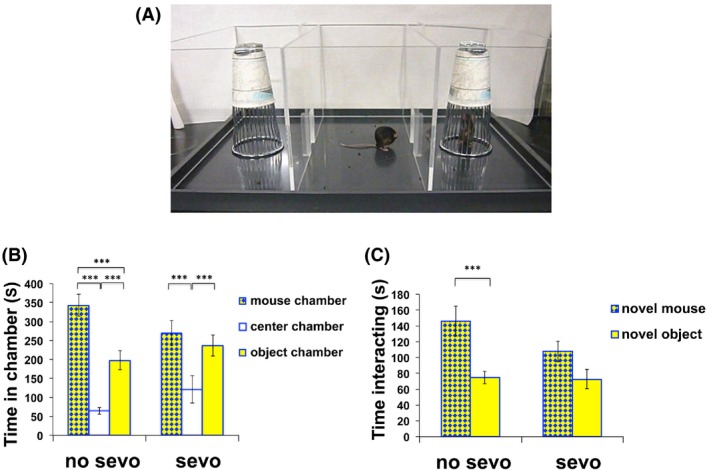
Neonatal sevo‐treated mice had a deficiency in social interaction behaviors as shown by three‐chamber social interaction. (A) A three‐chamber apparatus was used to examine social interaction as shown in the photograph. The apparatus is divided into an object chamber (left), a center chamber (center), and a novel mouse chamber (right). The doors on both the left and the right side of the center chamber are opened to allow the subject mouse to travel freely in between all three chambers. (B) The no sevo group spent significantly more time in the novel target mouse chamber compared to the object chamber, while the sevo group showed no such preference. Both groups of mice showed the least preference for the center chamber in which there was no novel target mouse or object present. Two‐way ANOVA, followed by Bonferroni posttest were used for statistical analysis, *F*_(1, 81)_ = 0, *P* > 0.05 for treatment; *F*_(2, 81)_ = 46.3, *P* < 0.0001 for chamber; *F*_(2, 81)_ = 2.9, *P* > 0.05 for interaction between chamber and treatment. Asterisk (***) denotes *P* < 0.001 for time in mouse versus object chamber for the no sevo group; *P* < 0.001 for time in mouse versus center and object versus center chamber for both the no sevo and the sevo groups. (C) A similar observation was made for the time the mice spent interacting with the novel target mouse or the novel object (sniffing and nose poking). The no‐sevo–treated mice spent significantly more time interacting with the novel target mouse than the novel object. Such an observation was not present in the sevo‐treated group. Two‐way ANOVA, followed by Bonferroni posttest were used for statistical analysis, *F*_(1, 53)_ = 1.3, *P* > 0.05 for treatment; *F*_(1, 53)_ = 14.6, *P* < 0.001 for subject/object; *F*_(1, 53)_ = 1.8, *P* > 0.05 for interaction between treatment and subject/object. Asterisk (***) denotes *P* < 0.001 for time spent interacting with subject/object for the no sevo group (*N *= 16 for no sevo; *N* = 13 for sevo).

We wanted to further understand whether the two groups of P7‐treated mice would differ in a social behavior paradigm that is self‐directed, without the physical elicitation from the novel target mouse. To address this question, we employed the three‐chamber social interaction paradigm (Nadler et al. 2004; Silverman et al. 2010). In this experimental setup, the subject mouse initiated the social approach, while the target novel mouse was confined under a wire pencil cup on one of the two sides of the three‐chamber apparatus (Fig. 6A). Confining the target novel mouse under the wire pencil cup prevented aggressive interaction between the two unfamiliar mice while providing olfactory, visual, auditory, and tactile contact.

In the no sevo group, mice spent significantly more time in the chamber with the confined novel mouse compared to the chamber with the novel object (an empty pencil cup; Fig. 6B; two‐way ANOVA, followed by Bonferroni posttests, *P* < 0.001). This behavior was not present for the sevo‐treated group, in which mice spent similar amount of time in the mouse chamber and the object chamber. Although the subject mouse was always placed in the center chamber at the initiation of the experiment, both no sevo and sevo groups showed the least interest in the center chamber compared to the two side chambers (Fig. 6B; two‐way ANOVA, followed by Bonferroni posttests, *P* < 0.001). While in the specific chambers, we made similar observations on the time that the no sevo versus sevo mouse spent interacting with the novel mouse or the novel object, such as nose poking or sniffing. The mice from the no‐sevo–treated group had significantly more interest in interacting with the novel mouse rather than the object, while the sevo‐treated group did not show a preference (Fig. 6C; two‐way ANOVA, followed by Bonferroni posttests, *P* < 0.001). Combining data from this experiment and reciprocal social interaction, we demonstrated that early‐life sevo treatment impacts social interaction later on in life.

### Communication

#### Olfactory

Olfactory cues are considered to be important for rodent communication such as mate choice, mother–infant bonding, aggressive interaction, territory recognition, and social bonding (Harrington 1976; Clutton‐Brock 1989; Ferguson et al. 2001; Broad et al. 2006; Stowers et al. 2013). Since the effect of early‐life general anesthetic exposure on communication is unclear, we examined this behavior by the olfactory habituation/dishabituation paradigm (Crawley et al. 2007).

**Figure 7:**
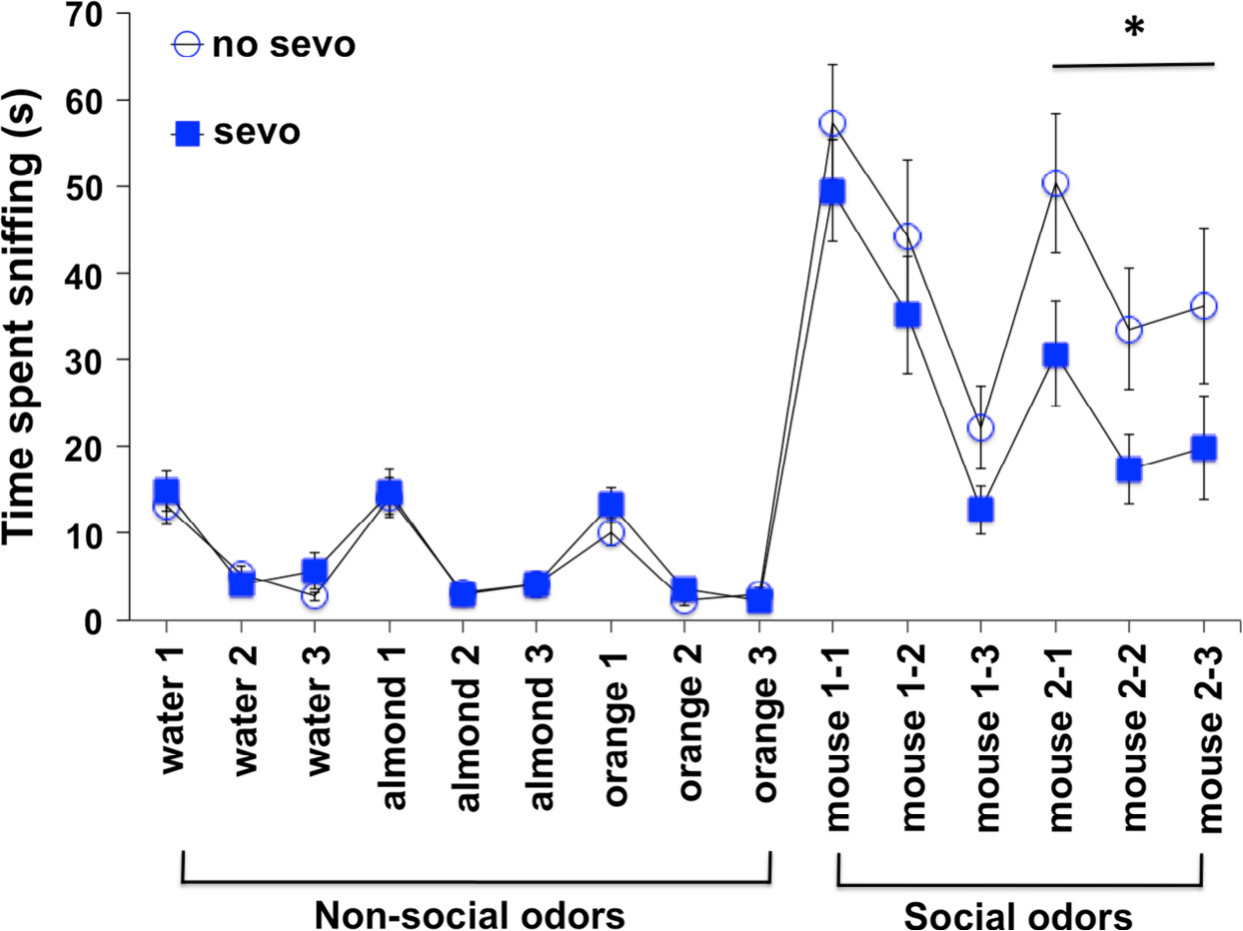
Both neonatal no‐sevo‐ and sevo‐treated mice showed no impairment in communication behavior based on olfactory habituation/dishabituation. Mice were able to habituate to the same scent when presented three consecutive times. This was shown by a significant decrease in the time spent sniffing from the first to the last presentation of the same scent. Repeated measure two‐way ANOVA resulted in *F*_(2, 66)_ = 30, *P* < 0.0001 for water; *F*_(2, 66)_ = 40, *P* < 0.0001 for almond; *F*_(2, 66)_ = 40, *P* < 0.0001 for orange; and *F*_(2, 66)_ = 40, *P* < 0.0001 for social 1 and *F*_(2, 66)_ = 5, *P* < 0.01 for social 2. There was no treatment difference on nonsocial and social 1 odor habituation. These mice were also able to dishabituate from old scents when new scents were presented. This was shown by a significant increase in every transition between the last presentation of the old scent to the first presentation of the new scent. A two‐way ANOVA resulted in *F*_(1, 66)_ = 31, *P* < 0.0001 for transition from water to almond; *F*_(1, 66)_ = 20, *P* < 0.0001 for transition from almond to orange; *F*_(1, 66)_ = 127, *P* < 0.0001 for transition from orange to social 1; and *F*_(1, 66)_ = 14, *P* < 0.001 for transition from social 1 to social 2. There was no treatment difference on odor dishabituation. However, sevo‐treated mice were observed to have a social interaction abnormality in this paradigm. There was a treatment difference on social 2 odor habituation, in which a repeated measure two‐way ANOVA resulted in *F*_(1, 66)_ = 4, *P* = 0.05 for treatment. Asterisk (*) denotes *P* = 0.05 for treatment effect on social 2 odors: mouse 2‐1, 2‐2, and 2‐3 (*N* = 18 for no sevo; *N* = 17 for sevo).

Mice were presented with three different nonsocial odors and two different social odors on cotton swabs. Both the sevo‐ and no‐sevo–treated groups were able to habituate to the same odor when it was presented three consecutive times. This is indicated by a significant decrease in time spent sniffing the cotton swabs of the same odor from the first to the third presentation (Fig. 7; repeated measure two‐way ANOVA, *P* < 0.0001 for time spent sniffing cotton swabs from the first to the third presentation of the odors: water, almond, orange, and social 1; repeated measure two‐way ANOVA, *P* < 0.01 for time spent sniffing cotton swabs from the first to the third presentation of the odor, social 2). There was no treatment difference on habituation for nonsocial and social 1 odors. Presentation of new odors elicited increased interest such that both groups of mice were able to dishabituate from the old scent to the new scent. This is indicated by a significant increase in time spent sniffing the cotton swab from the last presentation of the old odor to the first presentation of the new odor (two‐way ANOVA, *P* < 0.01–0.0001 for change of odor: transition from an old to a new odor). While both groups of mice showed similar behavior in olfactory cue habituation/dishabituation in nonsocial odors, we noticed a difference in their behavior toward the social odors. The sevo‐treated mice showed significantly less interest in sniffing compared to no‐sevo–treated mice when novel mouse 2 odors were presented (repeated measure two‐way ANOVA, *P* = 0.05 for treatment). These data further validate a deficiency in social interaction behavior in P7 sevo‐treated mice, an observation that has been recapitulated in two other behavioral paradigms in this current study: reciprocal social and three‐chamber social interaction.

## Discussion

In the United States, children under the age of 4 requiring single‐episode surgery‐related anesthetic exposure have the highest prevalence compared to two or more exposures (Wilder et al. 2009). Therefore, the effect of early‐life single‐episode anesthetic exposure on the long‐term functional consequence of brain development is particularly important. Our goal for this study is to capture this effect in our mouse‐model system using a clinically relevant anesthetic concentration and duration such that we can apply the results to better understand the risks in children. The major findings in this study are that P7 sevo‐exposed mice had impairments in cognition and social interaction behaviors later on in life.

It has been more than a decade since the first study in rodents demonstrated anesthetic neurotoxicity in the developing brain, resulting in impairment in cognitive function later on in life (Jevtovic‐Todorovic et al. 2003). However, the effects of single‐episode neonatal anesthetic exposure still face skepticism due to three major issues. First, animal studies lack a close mimic of anesthetic treatment parameters commonly applied to children among the single‐episode exposure group. Second, a more comprehensive understanding of the functional consequences of single‐episode exposure on different behavioral changes with disease implications has not been established. Third, it is difficult to dissociate general anesthesia from the underlying disease, coexisting conditions, or surgical procedures that require anesthesia. This study focuses on addressing these critical questions and they are discussed in detail in the following sections.

### Single‐episode anesthetic treatment in neonates

Skepticism toward the effect of neonatal single‐episode anesthetic exposure arises mainly due to inconsistency in experimental approaches, which ranges from anesthetic type to treatment duration and concentration. We chose to study only one type of anesthetic, sevoflurane, for several reasons. Unlike some other animal studies that used different anesthetic cocktails, treatment with sevo alone in this study provides a clear picture of its specific effects on the developing brain. Anesthetic cocktails that others have used to establish mouse models of neonatal anesthetic effects included nitrous oxide, which is often used as one of the induction agents, or ketamine, which is often used as procedural sedation or premedication in emergency rooms, but not as anesthetic maintenance for pediatric ambulatory surgeries (Alderson and Lerman 1994; Mellon et al. 2007; Lee et al. 2013). However, sevo is used for both anesthetic induction and maintenance, therefore it is often the main anesthetic agent during a pediatric surgical procedure (Goa et al. 1999).

To translate animal data and apply it to understand the clinical implications of general anesthesia, it is critical to adhere to treatment parameters commonly used among young children. Several of the most frequently performed procedures among children under the age of 4 are tonsillectomy, circumcision, hernia repair, and myringotomy with ear tube (Rabbitts et al. 2010). Children undergoing these types of surgeries are typically among the single‐episode anesthetic exposure group. The average duration of these types of surgeries is under 1 h and general anesthesia is most frequently used. However, among P7 rodent studies, exposure to anesthetics ranged from 4 to 6 h (Jevtovic‐Todorovic et al. 2003; Sanders et al. 2008; Loepke et al. 2009; Satomoto et al. 2009; Stratmann et al. 2009; Liang et al. 2010; Lee et al. 2014a,b). Longer exposure times ranging from 5 to 24 h were examined in monkeys (Zou et al. 2009, 2011; Brambrink et al. 2010; Paule et al. 2011). This is a 4‐ to 24‐fold increase in treatment duration compared to pediatric ambulatory surgeries and is not a realistic prediction for children among the single‐episode exposure group.

Our study approach consisted of 2 h of sevo treatment (Fig. 1). We did not reduce exposure to 1 h, but recognize that further reducing the duration would be a closer mimic to the common clinical surgeries among young children. Nevertheless, our approach is already the shortest duration currently used in animal studies, as we are unaware of any animal study with <2 h exposure duration. While others have used a range of 1–4% sevo on P7 rodents (Satomoto et al. 2009; Liang et al. 2010; Feng et al. 2012; Kato et al. 2013; Ramage et al. 2013; Shen et al. 2013b; Amrock et al. 2015), we used an average dosage of 2.38% of sevo. This concentration was less than the MAC for P7 mice, but was sufficient to keep the mice immobilized/unconscious throughout the duration of the treatment (Fig. 1). This further illustrates the deleterious effects of early‐life sevo, resulting in behavioral changes later on in life. Future works on fine‐tuning procedural approaches in order to establish the best rodent model for translational research are still necessary.

### The effect of neonatal single‐episode anesthetic exposure on different behavioral changes

One of the significances of the current study is that we demonstrated impairment in cognitive behavior by adhering our study approach closely to clinical scenarios and is based on two cognitive tasks that involve different brain regions. By the APA test, we demonstrated that P7 sevo‐treated mice had impairment in the hippocampus‐dependent spatial memory as early as periadolescent age (P27–P28; Fig. 3). Previous functional inactivation of the dorsal hippocampus blocked both the acquisition of spatial memories and the retrieval of long‐term spatial avoidance memories on the APA (Fenton et al. 1998; Cimadevilla et al. 2000; Kubik and Fenton 2005; Wesierska et al. 2005). Lesions in other brain regions such as the fornix or the anterior thalamus have also resulted in deficits in long‐term spatial memory (Aggleton et al. 1996); however, region‐specific tasks are needed to suggest their vulnerability to the effects of sevo during neonatal period. Since sevo targets receptors such as GABA_A_, glycine, and nicotinic acetylcholine that are ubiquitously expressed throughout the central nervous system (CNS) (Campagna et al. 2003; Rudolph and Antkowiak 2004), we wondered what other brain regions might be involved in neonatal sevo‐induced cognitive deficits. To approach this question, we examined the mice on a different cognition task, NOR (Fig. 4). This task was chosen specifically because unlike the APA, which is hippocampus‐dependent, this task examines recognition memory formation and is associated with the cortex, especially the medial temporal lobe and the thalamus (Brown and Aggleton 2001; Norman and Eacott 2004; Aggleton et al. 2011; Warburton and Brown 2015). We speculate that neonatal sevo‐induced cognitive impairment is associated with these brain regions, but would require future work at the anatomical, morphological, and electrophysiology level to support this.

Another significance of the current study is our investigation of the effects of neonatal sevo exposure on the development of neuropsychiatric‐like behavioral changes. Perturbation of the developing brain due to environmental insults is associated with numerous neurodevelopmental disorders. The exposure of human fetuses or neonatal rodents to antidepressants is paradoxically linked to changes in emotional behavior or the development of depression/anxiety later on in life (Ansorge et al. 2004; Hanley et al. 2013). Alcohol exposure during gestation is associated with a wide range of neurobehavioral disorders, including mood, cognition, and social interaction changes (Streissguth et al. 2004). Aside from toxins and drugs, early‐life abuse and neglect has been demonstrated to increase susceptibility to depression, schizophrenia, and anxiety‐related disorders (Batten et al. 2004; Phillips et al. 2005; Read and Hammersley 2005; Champagne and Curley 2009). Anesthetic exposure during neonatal surgical treatment is also an environmental insult to the developing brain. Therefore, we used a comprehensive approach by including social interaction (Figs. 5, 6), communication (Fig. 7), and repetitive behavior (Data S1) to better understand the neurodevelopmental changes of these mice. Among these behaviors, social interaction impairment associated with neonatal sevo treatment has been reported previously by another group (Satomoto et al. 2009). Using a caged social target in an open field apparatus, the group demonstrated that the no‐sevo–treated control group interacted significantly more with the social target compared to the sevo‐treated group. The novelty of the current study is, not only did we observed a deficit in social behavior in three different paradigms, reciprocal social interaction, three‐chamber social interaction, and olfactory habituation/dishabituation (Figs. 5, 6, 7); our study approach, compared to Satomoto et al., consisted of a threefold reduced treatment duration (2 hr, Lin et al., vs. 6 hr, Satomoto et al.) and a lower sevo dosage (2.38% sevo, Lin et al., vs. 3% sevo, Satomoto et al.).

Changes in social behavior could arise from an imbalance in excitatory/inhibitory neurotransmission, which has been shown in both mouse models of autism (Chao et al. 2010; Auerbach et al. 2011; Peca et al. 2011; Penagarikano et al. 2011; Han et al. 2012) and by optogenetic manipulation (Yizhar et al. 2011). It is temping to hypothesize that sevo's activation of GABA_A_ receptors in the developing brain may contribute to this imbalance. GABA exerts excitatory signaling in neurons during early development and then undergoes a switch to inhibition (Ben‐Ari et al. 2007). In rodents, the GABA switch extends over the entire second postnatal week and is completed in the third week. Developmental expression of the Cl^−^ extruder K^+^/Cl^−^ cotransporter, KCC2 is pivotal for the change from depolarizing to hyperpolarizing GABA_A_‐mediated action (Ben‐Ari et al. 2007). Exposure to sevo at P7 is concurrent with GABA's excitatory/inhibitory switch and the expression of KCC2. Whether excess activation of the GABA_A_ receptor by sevo during this critical developmental period has an effect on the expression of KCC2, is unknown. Future investigation on sevo's interference with the normal developmental excitatory/inhibitory switch would provide insight into the mechanisms underlying the observed functional changes of the brain.

### Dissociating general anesthesia from surgery

Animal models provide an ideal system to dissociate the underlying diseases and coexisting conditions that accompany surgery‐related anesthesia. Many studies used tail clamping of animals to establish the required MAC of anesthetics. Such approaches mimic clinical surgical procedures and are good animal models used to understand surgical‐related anesthesia. However, to understand the effect of anesthesia alone, we need to completely dissociate anesthesia from the act of surgery. Previously, two groups conducted learning and memory behavioral tests on neonatal sevo‐treated rats without tail clamping (Shih et al. 2012; Stratmann et al. 2014). However, rats in both groups were treated for 4 h with sevo ranging from 2.1% to 5.3%. Long treatment duration indicates potential physiologically intolerable toxicity; this resulted in decreased survival rates from 92% to 67% between the 2 and 4 h (Shih et al. 2012). The present study demonstrated for the first time a single‐episode, 2‐h treatment by sevo alone during the neonatal period impairs both social interaction and cognition.

## Concluding Remarks

We took the top‐down approach to study the effects of neonatal exposure to sevo by incorporating a battery of behavior paradigms. Our group showed for the first time that a single‐episode, 2‐h treatment of 2.3% sevo during the neonatal period impairs both social interaction and cognition later on in life. This study provides insight into the effects that clinically relevant neonatal anesthesia exposure have on the long‐term functional changes in the brain. With this information, we will then be able investigate the associated changes in electrophysiology, morphology, signal pathways, and molecular mechanisms. We are hopeful that our cumulative understanding will result in future therapeutic targets that may reverse the deleterious effects of early‐life anesthetic exposure on the developing brain.

## Conflict of Interest

None declared.

## Supporting information


**Data S1.** Repetitive behavior.
