# Supplementary figures and images for: Early‐life single‐episode sevoflurane exposure impairs social behavior and cognition later in life
Source: Brain Behav. 2016 Jul 4;6(9):e00514. doi: 10.1002/brb3.514 (PMC5036436; doi:10.1002/brb3.514)

Supplementary data:

A. Marble burying

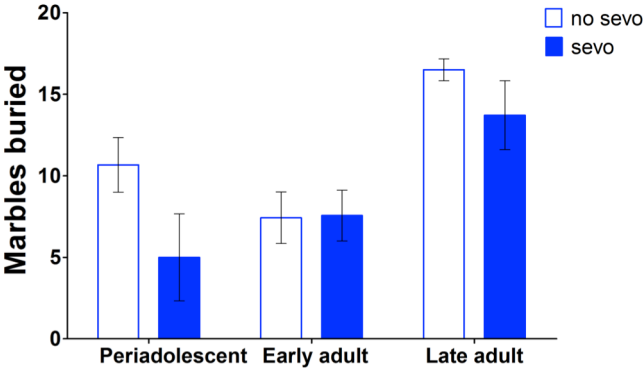

B. Repetitive self-grooming

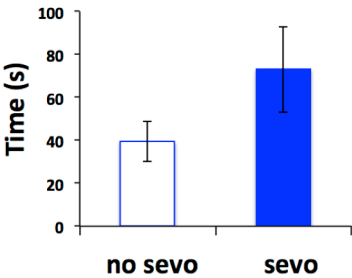

Supplement: Supplementary file 1 — Data S1. Repetitive behavior. [file BRB3-6-e00514-s001.pdf]
