# Supplementary material for: Early‐life single‐episode sevoflurane exposure impairs social behavior and cognition later in life
Source: Brain Behav. 2016 Jul 4;6(9):e00514. doi: 10.1002/brb3.514 (PMC5036436; doi:10.1002/brb3.514)
Supplement: Supplementary file 2 [file BRB3-6-e00514-s002.pdf]

Supplementary data

Repetitive Behavior

Repetitive and stereotypic movements are prominent features of both human and animal behaviors. A variety of abnormal repetitive behaviors are observed in neuropsychiatric disorders such as obsessive-compulsive disorder and autism spectrum disorder (Pichot, 1986). Since mice naturally exhibit self-grooming and digging behavior, we examined the P7 treated mice on repetitive self-grooming and marble burying paradigms (Thomas et al., 2009). The no sevo and sevo treated groups showed no differences in repetitive self-grooming and marble burying (unpaired Student's t-Test with Welch's correction). We concluded that one episode sevo exposure at P7 does not impact repetitive behavior later on in life.

Materials and Methods:

Repetitive movement

Repetitive grooming:

The mouse was transferred to a new cage with no bedding and habituated for 10 min. The mouse was then video recorded for 10 min and repetitive self-grooming behavior of the mouse was scored (McFarlane et al., 2008, Silverman et al., 2010).

Marble burying:

A mouse cage was prepared with fresh soft bedding up to 5 cm in height. 20 marbles were laid out on top of the bedding in 5 rows with 4 per row (Thomas et al., 2009). The mouse was then transferred to the cage with marbles and given 30 min to bury them. Unburied marbles were counted at the end of the experiment. Marbles were considered buried if they were half covered by the bedding.
